# Supplementary material for: Comprehensive approach to HIV/AIDS testing and linkage to treatment among men who have sex with men in Curitiba, Brazil
Source: PLoS One. 2021 May 10;16(5):e0249877. doi: 10.1371/journal.pone.0249877 (PMC8110178; doi:10.1371/journal.pone.0249877)
Supplement: Attachment — Submitted filename: REVIEW_PLOSdec2020.docx [file pone.0249877.s001.docx]

**Comprehensive approach to HIV/AIDS testing and linkage to treatment among men**

**who have sex with men in Curitiba, Brazil**

This is an interesting paper with important findings related to cost-effectiveness evaluation of several strategies used in Curitiba to expand access to HIV rapid testing in MSM and to improve the linkage of HIV positive MSM to health services and treatment.

However, to be published, in my opinion, substantial revisions are needed.

GENERAL OBSERVATIONS

1.I had some difficulties in order to understand the main objective of the paper and its specific objectives; in the abstract there are objectives defined but related to the project AHA, not to this paper; then, in the introduction (last paragraph) it is stated another different objective. Also these objectives are not related to the Title.

Reading the paper, I get the impression that the main objective of this paper is to compare the cost-effectiveness of several interventions/strategies used in Curitiba to expand access to HIV rapid testing in MSM and to improve the linkage of HIV positive MSM to health services and treatment. If it is the case, then the title should be different, namely it must reflect the type of approach used by the authors (health economic evaluation/ cost/ cost-effectiveness, etc..).

2. I had some difficulties in finding the most adequate Equator Guideline to review the article; however, I think that it should be the CHEERS Checklist- Items to include when reporting economic evaluations of health interventions. Consequently, the paper must be revised according to this Guideline.

SPECIFIC COMMENTARIES

ABSTRACT

Introduction. The authors must write in a very clear way the aim and the specific objectives of the paper (and not the objectives of the AHA)

Methods. Specify the type of study, the participants and statistical tests used to compare participant’s characteristics between groups.

Results. They should respond to specific objectives; it is not the case.

INTRODUCTION

In line 117 it will be interesting to have some figures about the prevalence/ incidence of HIV in Curitiba population if possible by sex and age and compared with the whole country.

In line 130, aim and specific objectives must be clearly defined (in order to be then compared with the results). If possible, specify the research question.

METHODS

This section must be reorganized, ideally following the structure bellow:

- Target population and subgroups
- Setting and location
- Study perspective and its relation to the costs being evaluated.
- Description of the interventions or strategies being compared and

state why they were chosen.

- Time horizon
- Choice of health outcomes
- Measurement of effectiveness
- Estimating resources and costs
- Statistical Analysis (with the tests used) and why they were chosen

In lines, 138-141, separate the type of study, from the variables.

In lines 235-241 the description of variables is not a statistical analysis.

In line 241 the authors stated they used frequencies and medians; its ok; however, in the results (footnotes in Tables 2 and 3) they refer to statistical tests; these tests must be described in the statistical analysis section.

Lines 242-245 are not related to statistical analysis.

RESULTS

The Results section must follow CHEERS guidelines and must be coherent with the specific objectives.

Table 2 in footnote 6 it is stated that: Statistical tests were performed for the difference of proportions (z test and Fisher's test) in pairs for the strategies - COA and NGO; COA and MTU; NGOs and MTU - only for COA and NGO, *the result was not significant.*

The sentence: *the result was not significant* is not adequate; what result? What is being compared? What is the significant level?

Tests must be described in Methods section; and in the results we have to understand which result was not significantly different from another one. Because we are comparing groups. More rigour is needed.

Table 3. Footnote. Same observation.

In lines 304-312. In the sentence comparing groups include p values inside brackets

Table 4. The title is related to COST-EFFECTIVENESS. It is the first time we are faced with this expression that has a very powerful meaning in economic evaluations of health interventions

The authors must explain in the Methods section what type og cost-effectiveness approach they are using and why?

Figures 2 and 3 are not very clear from the graphical point of view. I think that tables with figures are more frequently used in this context.

DISCUSSION AND CONCLUSION: will depend a lot on the revisions made to methods and results.
