# Supplementary material for: Comprehensive approach to HIV/AIDS testing and linkage to treatment among men who have sex with men in Curitiba, Brazil
Source: PLoS One. 2021 May 10;16(5):e0249877. doi: 10.1371/journal.pone.0249877 (PMC8110178; doi:10.1371/journal.pone.0249877)
Supplement: Attachment — Submitted filename: rebuttal_letter.docx [file pone.0249877.s002.docx]

Rio de Janeiro, February 18th 2021

Dear Editors,

In compliance with the observations described by the academic editors and reviewers, please find bellow the responses and changes made to the manuscript titled “Comprehensive approach to HIV/AIDS testing and linkage to treatment among men who have sex with men (MSM) in Curitiba, Brazil”.

**Reviewer #1:**

This is a useful study looking at various HIV testing/screening methods in Brazil. However, additional clarification around each strategy and the methodology would help the reader more accurately assess the effectiveness of each. The economic evaluation could be enhanced.

**Introduction**
Paragraph starting with line 101: The incidence rates cited are now 6 years old. How have trends changed more recently with the introduction of PrEP?

**Response:** the reference has been updated.

**Methods**
The methods would be more clear if the three comparisons were stated straight away. It is not clear what is being compared. From table 1, it appears that the MTU is being compared with fixed services at the COA and NGO. However, the text then introduces self-testing. How does the self-testing fit? Is it a separated comparison group or is it a function of one or all of the aforementioned groups?

**Response:** this section was re-written. The self -testing strategy is not being compared with the other three strategies (COA, NGO and MTU).

Other relevant information that needs to be more clear: did all strategies occur at the same time, or were they simultaneous? Did all allow all ages? Did all recruit MSM equally, or did particular strategies target a more general LGBT audience?

**Response:** please see time horizon and target population. All strategies targeted the same population equally – MSM, however the e-testing (self-testing) targeted MSM aged 18 and above and the other three strategies, target MSM aged 14 and above.

Cost study-did the cost study cover different dates/time period than the testing data? If not, please clarify (the dates appear to overlap in table 4, but this is not mentioned in the methods). If so, I suggest revising to have costs and test dates align.
**Response:** please see time horizon. The cost study collected retrospective from each testing outlet covering a 12-month period (March 2015 to February 2016).

**Results**
Self-testing
Was there an attempt to record the results from these tests?
**Response:** the results were collected and reported however there is no comparison with the other three strategies

**Discussion**
The discussion about cost seems to be missing text? Line 425 introduces this topic by saying that self-testing is ranked second. There is no other discussion of costs. This should be added. Also, the conclusion that self-testing is effective is not supported by the data in this analysis since we don’t know how many people actually used the tests that were delivered.

**Response:** this paragraph has been re-written

**Reviewer #2:**

The paper must be substantially improved in order to be published. Please see specific the comments in the attach file.
The paper must follow some EQUATOR guidelines; in my opinion the CHEERS is the most adequate.

**General Observations**

1.I had some difficulties in order to understand the main objective of the paper and its specific objectives; in the abstract there are objectives defined but related to the project AHA, not to this paper; then, in the introduction (last paragraph) it is stated another different objective. Also these objectives are not related to the Title.

Reading the paper, I get the impression that the main objective of this paper is to compare the cost-effectiveness of several interventions/strategies used in Curitiba to expand access to HIV rapid testing in MSM and to improve the linkage of HIV positive MSM to health services and treatment. If it is the case, then the title should be different, namely it must reflect the type of approach used by the authors (health economic evaluation/ cost/ cost-effectiveness, etc..).

**Response:** revisions made

2. I had some difficulties in finding the most adequate Equator Guideline to review the article; however, I think that it should be the CHEERS Checklist- Items to include when reporting economic evaluations of health interventions. Consequently, the paper must be revised according to this Guideline.

**Response:** revisions made

**Specific Commentaries**

**Abstract**

Introduction. The authors must write in a very clear way the aim and the specific objectives of the paper (and not the objectives of the AHA)

Methods. Specify the type of study, the participants and statistical tests used to compare participant’s characteristics between groups.

Results. They should respond to specific objectives; it is not the case.

**Response:** the abstract was rewritten.

**Introduction**

In line 117 it will be interesting to have some figures about the prevalence/ incidence of HIV in Curitiba population if possible by sex and age and compared with the whole country.

**Response:** information added to the manuscript

In line 130, aim and specific objectives must be clearly defined (in order to be then compared with the results). If possible, specify the research question.

**Response:** the aims and objectives were rewritten.

**Methods**

This section must be reorganized, ideally following the structure bellow:

- Target population and subgroups
- Setting and location
- Study perspective and its relation to the costs being evaluated.
- Description of the interventions or strategies being compared and

state why they were chosen.

- Time horizon
- Choice of health outcomes
- Measurement of effectiveness
- Estimating resources and costs
- Statistical Analysis (with the tests used) and why they were chosen

**Response:** This section has been re-written

In lines, 138-141, separate the type of study, from the variables.

**Response:** it was re-written

In lines 235-241 the description of variables is not a statistical analysis.

**Response:** correction made – description was moved to “Choice of health outcomes” within methods section

In line 241 the authors stated they used frequencies and medians; its ok; however, in the results (footnotes in Tables 2 and 3) they refer to statistical tests; these tests must be described in the statistical analysis section.

**Response:** has been rewritten and is in the statistical analysis section within methods section

Lines 242-245 are not related to statistical analysis.

**Response:** correction made – description was moved to “Choice of health outcomes” within methods section

**Results**

The Results section must follow CHEERS guidelines and must be coherent with the specific objectives.

**Response:** revision was made in both sections in order to adjust coherency with the objectives. We did not follow Cheers guidelines as this paper is not an economic evaluation, even though there is cost analysis included in it.

Table 2 in footnote 6 it is stated that: Statistical tests were performed for the difference of proportions (z test and Fisher's test) in pairs for the strategies - COA and NGO; COA and MTU; NGOs and MTU - only for COA and NGO, *the result was not significant.*

The sentence: *the result was not significant* is not adequate; what result? What is being compared? What is the significant level?

Tests must be described in Methods section; and in the results we have to understand which result was not significantly different from another one. Because we are comparing groups. More rigour is needed.

**Response:** the footnote was rewritten and the tests performed were described in the statistical analysis section within methods

Table 3. Footnote. Same observation.

**Response:** the footnote was rewritten and the tests performed were described in the statistical analysis section within methods

Table 4. The title is related to COST-EFFECTIVENESS. It is the first time we are faced with this expression that has a very powerful meaning in economic evaluations of health interventions

The authors must explain in the Methods section what type of cost-effectiveness approach they are using and why?

**Response:** this section has been re-written with appropriated wording

Figures 2 and 3 are not very clear from the graphical point of view. I think that tables with figures are more frequently used in this context.

**Response:** the figures were transformed into tables

My co-authors and I would like to thank you for your revision on this manuscript.

Regards

Vanda Cota
