# Supplementary material for: Comprehensive approach to HIV/AIDS testing and linkage to treatment among men who have sex with men in Curitiba, Brazil
Source: PLoS One. 2021 May 10;16(5):e0249877. doi: 10.1371/journal.pone.0249877 (PMC8110178; doi:10.1371/journal.pone.0249877)
Supplement: Attachment — Submitted filename: rebuttal_letter.docx [file pone.0249877.s003.docx]

Rio de Janeiro, March 23rd 2021

Dear Editors,

Thank you again for reviewing the manuscript titled “Comprehensive approach to HIV/AIDS testing and linkage to treatment among men who have sex with men (MSM) in Curitiba, Brazil”. We have addressed all the comments made.

Methods Section, Statistical Analysis: The first paragraph must be corrected as suggested below:

 "In the analyzes, absolute frequencies, ......, as well as unilateral statistical tests for the difference in proportions - z test and/or Fisher test, depending on the number of expected observations per cell."

**Response:** Changes made.

In the third paragraph don’t include table numbers (Table 3) because these tables are in the results.

**Response:** Changes made.

Results section: Footnotes in Table 3 need to be revised.

Footnote 5- According to test, the proportion of the NGO (68.2%) is not significantly greater than that of the MTU (67.5%).

Footnote 6- According to the test, the proportion of the COA (69.8%) is not significantly lower than that of the NGO (80.0%).

**Response:** You mentioned table 3, but the notes refer to table 2. Changes made on table 2.

In table 3 replace average age by median age to be consistent with the sentence in the paragraph below the table.

**Response:** Changes made.

Regards

Vanda Cota
